# Thalassaemia is paradoxically associated with a reduced risk of in‐hospital complications and mortality in COVID‐19: Data from an international registry

**DOI:** 10.1111/jcmm.17026

**Published:** 2022-03-30

**Authors:** Ibrahim El‐Battrawy, Filomena Longo, Iván J. Núñez Gil, Mohammad Abumayyaleh, Barbara Gianesin, Vicente Estrada, Álvaro Aparisi, Ramón Arroyo‐Espliguero, Manuela Balocco, Susanna Barella, Andrea Beccaria, Federico Bonetti, Maddalena Casale, Elisa De michele, Anna Rita Denotti, Carmelo Fidone, Monica Fortini, Maria Rita Gamberini, Giovanna Graziadei, Roberto Lisi, Antonella Massa, Alessia Marcon, Bryan Rubinski, Maurizio Miano, Irene Motta, Valeria Maria Pinto, Alberto Piperno, Raffaella Mariani, Maria Caterina Putti, Alessandra Quota, Michela Ribersani, Marco Marziali, Domenico Roberti, Rosamaria Rosso, Immacolata Tartaglione, Angelantonio Vitucci, Vincenzo Voi, Marco Zecca, Rodolfo Romero, Charbel Marouneld, Inmaculada Fernández‐Rozas, Carolina Espejo, Wulandewi Marhaeni, Marcos Garcia Aguado, Maria Domenica Cappellini, Silverio Perrotta, Lucia De Franceschi, Antonio Piga, Gian Luca Forni, Ibrahim Akin

**Affiliations:** ^1^ University of Mannheim Mannheim Germany; ^2^ Department of Clinical and Biological Sciences University of Turin Turin Italy; ^3^ Hospital Clínico San Carlos Universidad Complutense de Madrid Instituto de Investigación Sanitaria del Hospital Clínico San Carlos (IdISSC) Madrid Spain; ^4^ ForAnemia Foundation Genoa Italy; ^5^ Hospital Clínico Universitario de Valladolid Valladolid Spain; ^6^ Hospital Universitario Guadalajara Guadalajara Spain; ^7^ E.O. Ospedali Galliera Genoa Italy; ^8^ SSD Talassemia. Ospedale Pediatrico Microcitemico A. Cao Cagliari Italy; ^9^ Istituto Giannina Gaslini Genoa Italy; ^10^ Fondazione IRCCS Policlinico San Matteo Pavia Italy; ^11^ Dipartimento della Donna, del Bambino e di Chirurgia Generale e Specialistica Università degli Studi della Campania Luigi Vanvitelli Naples Italy; ^12^ AOU OO.RR. San Giovanni di Dio Ruggi d'Aragona Salerno Italy; ^13^ Unità operativa semplice Studio Emoglobinopatie Simt Ragusa Italy; ^14^ Azienda Ospedaliero‐Universitaria S.Anna di Ferrara Ferrara Italy; ^15^ Department of Clinical Sciences and Community Health Università degli Studi di Milano Milan Italy; ^16^ Fondazione IRCCS Ca’ Granda Ospedale Maggiore Policlinico Milan Italy; ^17^ Azienda Ospedaliera di Rilievo Nazionale e di Alta Specializzazione Garibaldi Catania Italy; ^18^ Ospedale Giovanni Paolo II Olbia Italy; ^19^ ASST‐Monza, S.Gerardo Hospital University of Milano‐Bicocca Monza Italy; ^20^ Azienda Ospedale Università Padova Padua Italy; ^21^ UOS Talassemia P.O. Vittorio Emanuele Gela (Caltanissetta) Italy; ^22^ Policlinico Umberto I Rome Italy; ^23^ Azienda Ospedaliero‐Universitaria Policlinico "Vittorio Emanuele" Catania Italy; ^24^ Azienda Ospedaliera Universitaria Policlinico Bari Italy; ^25^ Policlinico GB Rossi Verona Italy; ^26^ Emergency Department Hospital Universitario Getafe Getafe (Madrid) Spain; ^27^ HOSPITAL UNIVERSITARIO LA PAZ Instituto de InvestigaciÃ³n Hospital Universitario La Paz (IdiPAZ) Madrid Spain; ^28^ Hospital Universitario Severo Ochoa Cardiology LeganÃ Spain; ^29^ Hospital Universitario PrÃncipe de Asturias Madrid Spain; ^30^ Pediatrics Department Hematology‐Oncology Division Faculty of Medicine Lambung Mangkurat University Banjarmasin Indonesia; ^31^ Cardiology Department Hospital Puerta de Hierro de Majadahonda Majadahonda, Madrid Spain; ^32^ Department of Cardiology BG Universitätsklinikum Bochum, Ruhr‐Universität Bochum Bochum Germany

**Keywords:** COVID‐19, mortality, SARS‐CoV‐2, thalassaemia

## Abstract

Although numerous patient‐specific co‐factors have been shown to be associated with worse outcomes in COVID‐19, the prognostic value of thalassaemic syndromes in COVID‐19 patients remains poorly understood. We studied the outcomes of 137 COVID‐19 patients with a history of transfusion‐dependent thalassaemia (TDT) and transfusion independent thalassaemia (TIT) extracted from a large international cohort and compared them with the outcomes from a matched cohort of COVID‐19 patients with no history of thalassaemia. The mean age of thalassaemia patients included in our study was 41 ± 16 years (48.9% male). Almost 81% of these patients suffered from TDT requiring blood transfusions on a regular basis. 38.7% of patients were blood group O. Cardiac iron overload was documented in 6.8% of study patients, whereas liver iron overload was documented in 35% of study patients. 40% of thalassaemia patients had a history of splenectomy. 27.7% of study patients required hospitalization due to COVID‐19 infection. Amongst the hospitalized patients, one patient died (0.7%) and one patient required intubation. Continuous positive airway pressure (CPAP) was required in almost 5% of study patients. After adjustment for age‐, sex‐ and other known risk factors (cardiac disease, kidney disease and pulmonary disease), the rate of in‐hospital complications (supplemental oxygen use, admission to an intensive care unit for CPAP therapy or intubation) and all‐cause mortality was significantly lower in the thalassaemia group compared to the matched cohort with no history of thalassaemia. Amongst thalassaemia patients in general, the TIT group exhibited a higher rate of hospitalization compared to the TDT group (*p* = 0.001). In addition, the rate of complications such as acute kidney injury and need for supplemental oxygen was significantly higher in the TIT group compared to the TDT group. In the multivariable logistic regression analysis, age and history of heart or kidney disease were all found to be independent risk factors for increased in‐hospital, all‐cause mortality, whereas the presence of thalassaemia (either TDT or TIT) was found to be independently associated with reduced all‐cause mortality. The presence of thalassaemia in COVID‐19 patients was independently associated with lower in‐hospital, all‐cause mortality and few in‐hospital complications in our study. The pathophysiology of this is unclear and needs to be studied in vitro and in animal models.

## 1 INTRODUCTION

Since publication of the first reports on COVID‐19, the highest incidence and mortality rates have been reported in the USA, Italy, Spain, China and France,^1^ with nearly 2.2 million deaths and more than 103,000,000 infections having been reported worldwide to date.^2^, ^3^, ^4^, ^5^ Although patients may present with symptoms such as fever, myalgia, fatigue, dry cough and/or diarrhea,^6^ asymptomatic patients are not rare. Different models have been developed to date to analyse the severity of COVID‐19, with numerous co‐factors having been reported as predictors of worse outcomes such as arterial hypertension, diabetes mellitus, heart disease, acute and chronic kidney disease, chronic pulmonary disease, advanced age, immunosuppression and inherited haemoglobin disorders.^7^, ^8^, ^9^, ^10^, ^11^ Few data have been published to date on COVID‐19 and thalassaemia syndrome and the existing data are still controversial due to the small number of patients included.^8^, ^12^, ^13^, ^14^

Thalassaemia patients may be at high risk for infectious complications leading to increased mortality and morbidity. This may be due to splenectomy, adrenal insufficiency, stem cell transplantation and/or stress erythropoiesis.^15^ Despite advances in the understanding of the pathophysiology of thalassaemia over the last decade, management of this disease has remained largely unchanged over the years.^16^

In this present study, we described the clinical characteristics and outcomes of a cohort of COVID‐19 patients suffering from concomitant thalassaemia using data from a large, international observational study and compared them to a matched cohort with no history of thalassaemia.

## 2 METHODS

### 2.1 Study design and participants

Data from the HOPE‐COVID‐19 (Health Outcome predictive Evaluation for COVID‐19, NCT04334291) and from the observational study from the ‘Società Italiana per le Talassemie ed Emoglobinopatie’ (SITE), EMO AER COVID‐19 (Observational Study for Patients With Hemoglobinopathies and Rare Inherited Anemia and Covid 19, NCT04746066) were extracted and designed as a retrospective registry without any financial support. An online database was built and completed by each participating centre (www. HopeProjectMD.com, http://www.site‐italia.org/2020/covid‐19.php). We analysed all 137 thalassaemia patients identified from the beginning of March to the 30th of November 2020. No patients were excluded. The study was approved by the Ethics Committees in all of the centres involved (Banjarmasin, Bari, Cagliari, Catania, Ferrara, Gela, Genoa, Getafe, Guadalajara, Legan, Madrid, Mannheim, Milan, Monza, Naples, Olbia, Padua, Pavia, Ragusa, Rome, Salerno, Turin, Valladolid and Verona).

### 2.2 Thalassaemia

The data set included different clinical forms of β‐thalassaemia, including the co‐inheritance of β‐thalassaemia with haemoglobin E resulting in haemoglobin E/β‐thalassaemia. Patients were managed according to the current recommendations for the treatment of thalassaemia.^14^

### 2.3 Outcomes

The primary outcome was in‐hospital, all‐cause mortality. In addition, in‐hospital complications including admission to an intensive care unit (ICU), as well as the type of respiratory support needed (nasal cannula, non‐invasive mechanical ventilation, invasive mechanical ventilation or invasive mechanical support), were also evaluated in the outcome analysis.

### 2.4 Data extraction

Epidemiological, clinical and outcome data were collected from the electronic medical records. The confidentiality of patient data was protected by storing them in an anonymous manner on a locked, password‐protected computer and/or website. Blood test results, biochemical tests and radiology images were also extracted from the electronic medical records. Comorbidities such as dyslipidemia, diabetes mellitus, obesity, current smoking, kidney disease, lung disease, heart disease, splenectomy and stem cell transplantation were recorded at the time of admission. Data regarding disease management and treatment were also extracted and included the use of different drugs and/or respiratory support. We first identified all COVID‐19 patients with a history of thalassaemia. Then, we compared this cohort with a COVID‐19 cohort matched according to age, gender, diabetes, kidney disease, cardiac disease and pulmonary disease with no history of thalassaemia. For the Cox regression analysis, the entire COVID‐19 cohort consisting of 6109 COVID‐19 patients recruited from the participating centres was included.

### 2.5 Statistical analysis

Continuous variables with a normal distribution are shown as mean ± standard deviation, continuous variables with a non‐normal distribution are shown as a median (interquartile range) and categorical variable are shown as a frequency (%). The Kolmogorov‐Smirnov test was used to assess normal distribution. Continuous variables with normal and non‐normal distributions were compared using a Student's *t* test and the Mann‐Whitney *U* test, respectively. Categorical variables were compared with a Chi‐squared test or Fisher's exact test. A *p* value less than 0.05 was considered as statistically significant. We performed propensity score matching (PSM 1:1; 0.001 tolerance for the 127 PSM, without replacement, nearest neighbour) was performed in order to adjust for the different clinical profiles observed and obtain balanced pairs. The following variables were included age, gender, diabetes, heart disease and pulmonary disease. Statistical analysis was performed using SPSS statistics v25.0 (SPSS, Inc.,). Cox logistic regression analysis was performed in order to identify possible independent risk factors. Factors with a *p*‐value <0.05 were included in a multivariable logistic regression analysis.

## 3 RESULTS

### 3.1 Baseline characteristics

The mean age of thalassaemia patients was 41 ± 16 years (48.9% male). Almost 81% of these patients were found to require blood transfusions on a regular basis. More than one third of patients (53 patients/38.7%) had the blood group O. Cardiac iron overload was documented in only 6.8% of patients, whereas liver iron overload was documented in 35% of patients. 10.2% of patients had a history of diabetes mellitus, 8.1% obesity, 8.8% pulmonary disease, 4.1% pulmonary hypertension and 19.7% cardiac disease. With regard to thalassaemia treatments, splenectomy was documented in one 40% of patients, hydroxyurea therapy in 5.9% and chelation therapy in 117 patients. The baseline characteristics of the study patients are listed in Table 1. Table S1 presents the cohort of 134 patients separated into a TIT and a TDT group. The information regarding a history of transfusion was missing in three patients. Patients in the TIT group were in general older than in the TDT group (54.7 ± 18.6 years versus 38 ± 14, *p* < 0.001) and hospitalized more often (*p* = 0.001). Symptoms such as dyspnoea, fatigue, fever and cough were significantly more prevalent in the TIT group compared to TDT group. In‐hospital complications were similar between groups except with regard to the need for supplemental oxygen and acute kidney injury, both of which were more common in the TIT group.

**TABLE 1:** Characteristics of all thalassaemia patients at Baseline

Characteristic	*N* = 137
Age—yr mean ± SD ç	41 ± 16
Male sex—no. (%)	67/137 (48.9)
**Thalassaemia type**—**no. (%)**	
TIT	22/134 (16.1)
TDT	112/134 (81.8)
**Blood Group**—**no. (%)**	
A Rh‐	2/109 (1.5)
A Rh+	37/109 (27)
AB Rh+	3/109 (2.2)
B Rh+	14/109 (10.2)
0 Rh‐	4/109 (2.9)
0 Rh+	49/109 (35.8)
**Iron load**	
Serum Ferritin mg/l (median(min‐max))	682 (27–8140)
Liver MRI T2 ms (mean ± SD)	10.98 ± 7.4
Heart MRI T2 ms (mean ± SD)	33.93 ± 10.2
LIC (mg Fe/g d.w.) (median(min‐max))	2.94 (1.05–127.2)
Cardiac iron overload—no. (%)	5/74 (6.8)
Hepatic iron overload—no. (%)	28/80 (35)
**Therapy**—**no. (%)**	
**Chelation therapy**	117
DFO	16 (13.7)
DFP	20 (17.1)
DFX	59 (50.4)
DFO+DFX	2 (1.7)
DFP+DFO	7 (6)
DFP+DFX	3 (2.6)
No therapy	10 (8.5)
**HU therapy**	6/101 (5.9)
**Chronic conditions**—**no. (%)**	
Diabetes Mellitus	14/137 (10.2)
Obesity	11/136 (8.1)
Renal insufficiency	7/137 (5.1)
Lung disease	12/137 (8.8)
Cardiac disease	27/137 (19.7)
Pulmonary hypertension	5/123 (4.1)
Liver disease	19/137 (13.9)
Previous HCV hepatitis	3/123 (2.4)
Hypothyroidism	20/137 (14.6)
Hypogonadism	17/123 (13.8)
Osteoporosis	31/123 (25.2)
G6PD deficiency	4/122 (3.3)
Splenectomy	50/127 (39.4)

Abbreviations: CPAP, Continuous positive airway pressure; CT, Computer tomography; DFO, Deferoxamine; DFP, Deferiprone; DFX, Deferasirox; G6PD, Glucose‐6‐phosphate dehydrogenase; HCQ, Hydroxychloroquine; HCV, Hepatitis virus C; HU, Hydroxyurea; ICU, Intensive care unit; IgG, Immunoglobulin g; LIC, Liver iron concentrations; LMWH, Low‐molecular‐weight heparin; MEEX, Manual erythron‐exchange; MRI, Magnet resonance imaging; TDT, Transfusion dependency; TIT, Non‐transfusion dependency.

### 3.2 In‐hospital complications

27.7% of the thalassaemia patients were hospitalized due to COVID‐19 and 18% of patients required admission to an intensive care unit (ICU). The main presenting symptoms were as follows: dyspnoea, rhinorrhoea, fatigue, anosmia or dysgeusia, pain, headache, sore throat and/or diarrhoea. Fever was documented in almost 51% of patients. 65% of patients exhibited changes in the thoracic CT imaging. 8,1% of patients received hydroxychloroquine, 6,7% lopinavir or ritonavir and 10% glucocorticoids. Antibiotics were prescribed to 17.6% of patients. Blood transfusions were required in 5.5% of cases. Non‐invasive respiratory support with continuous positive airway pressure (CPAP) therapy was required in 5.2% of cases and invasive mechanical ventilation in 0.7%. In general, supplemental oxygen support was required in 10% of patients. Overall, only one study patient (0.7%) died. Complications, treatment regimens and outcomes are listed in Table 2.

**TABLE 2:** Complication, therapeutic procedures and outcome of thalassaemia patients during COVID‐19

Hospitalized	38/137 (27.7)
ICU Admission—no. (%)	25/137 (18.2)
**Symptomatic**—**no. (%)**	
Dyspnoea	27/137 (19.7)
Rhinorrhoea	29/137 (21.2)
Fatigue	24/137 (17.5)
Anosmia / Dysgeusia	35/137 (25.5)
Pain	48/137 (35)
Headache	33/137 (24.1)
Sorethroat	35/137 (25.5)
Fever	70/137 (51.1)
Cough	63/137 (46)
Diarrhoea	13/137 (9.5)
X‐ray abnormality—no. (%)	25/40 (62.5)
CT abnormality—no. (%)	15/23 (65.2)
**Complications and procedures**—**no. (%)**	
Co‐infection	3/137 (2.2)
Acute kidney injury	5/137 (3.6)
Pulmonary embolism	1/137 (0.7)
Required oxygen support	14/137 (10.2)
CPAP	7/135 (5.2)
Invasive mechanical ventilation	1/136 (0.7)
**Therapeutic procedures**—**no. (%)**	
HCQ	11/136 (8.1)
Interleukin−1 receptor antagonist	1/127 (0.8)
Lopinavir/ritonavir	9/135 (6.7)
HCQ, tocilizumab and lopinavir/ritonavir	3/135 (2.2)
Glucocorticoid	14/135 (10.4)
Antibiotic therapy	24/136 (17.6)
LMWH	15/135 (11.1)
Remdesivir	3/127 (2.4)
Blood transfusion	7/127 (5.5)
MEEX	1/122 (0.8)
**Outcome**—**no. (%)**	
Recovered	136/137 (99)
Dead	1/136 (0.7)

Abbreviations: CPAP, Continuous positive airway pressure; CT, Computer tomography; DFO, Deferoxamine; DFP, Deferiprone; DFX, Deferasirox; G6PD, Glucose‐6‐phosphate dehydrogenase; HCQ, Hydroxychloroquine; HCV, Hepatitis virus C; HU, Hydroxyurea; ICU, Intensive care unit; IgG, Immunoglobulin g; LIC, Liver iron concentrations; LMWH, Low‐molecular‐weight heparin; MEEX, Manual erythron‐exchange; MRI, Magnet resonance imaging; TDT, Transfusion dependency; TIT, Non‐transfusion dependency.

### 3.3 Comparison of outcomes in patients with and without a history of thalassaemia

In the propensity score matching, 127 COVID‐19 patients with concomitant thalassaemia were matched to 127 patients suffering from COVID‐19 with no history of thalassaemia. Patients with no history of thalassaemia exhibited significantly higher rates of hospitalization than those with a history of thalassaemia as seen in Table 3. Cardiac disease, kidney disease, pulmonary disease and cardiovascular risk factors such as diabetes mellitus were similar between both groups. Amongst thalassaemia patients, 14.1% suffered from liver disease compared to 3.9% without thalassaemia; *p* < 0.001. In general, COVID‐19 symptoms including dyspnoea, fatigue, sore throat, fever, cough and diarrhoea were significantly less prevalent in patients with a history of thalassaemia compared to those with no history of thalassaemia. Complications including anaemia at admission (4.6% versus 23.6%), acute kidney injury (3.1% vs. 18.1%), need for supplemental oxygen support (9.4% vs. 74.8%), CPAP therapy (5.5% vs. 22.8%) and death (0% vs. 25%) were significantly less prevalent in thalassaemia patients compared to the patients with no history of thalassaemia. Consequently, the use of various drug treatments such as hydroxychloroquine, hydroxychloroquine plus lopinavir or ritonavir, hydroxychloroquine plus tocilizumab plus lopinavir/ritonavir, glucocorticoid therapy and antibiotics were used at significantly lower rates in thalassaemia patients.

**TABLE 3:** Characteristics of all Patients at Baseline, complication, therapeutic procedures and outcome after propensity score matching

Characteristic	Matched *n* = 254		
	Patients without Thalassaemia *n* = 127	Patients with Thalassaemia *n* = 127	*p* value
Age—year mean ± SD	42.8 ± 14.8	43.8 ± 16.1	0.63
Male sex—no. (%)	60 (47.2)	58 (45.6)	0.80
**Hospitalization**—**no. (%)**	125 (98.4)	33 (25.9)	**<0.001**
**Chronic conditions**—**no. (%)**			
Diabetes Mellitus	12 (9.4)	11 (8.6)	0.82
Renal insufficiency	3 (2.3)	7 (5.5)	0.19
Lung disease	8 (6.3)	12 (9.4)	0.35
Cardiac disease	30 (23.6)	25 (19.6)	0.44
Liver disease	5 (3.9)	18 (14.1)	**<0.01**
**Symptomatic**—**no. (%)**			
Dyspnoea	93 (73)	25 (19.6)	**<0.001**
Fatigue	64 (50.3)	22 (17.3)	**<0.01**
Anosmia/Dysgeusia	23 (18.1)	34 (26.7)	0.17
Pain	47 (37)	46 (36.2)	0.56
Sorethroat	29 (22.8)	34 (26.7)	**<0.001**
Fever	106 (83.4)	67 (52)	**<0.001**
Cough	100 (78.7)	60 (47.2)	**<0.001**
Diarrhoea	25 (19.7)	12 (9.4)	**0.01**
**Radiological findings**—**no. (%)**			
**Complications and procedures**—**no. (%)**			
Anaemia	30 (23.6)	6 (4.6)	**0.03**
Acute kidney injury	23 (18.1)	4 (3.1)	**<0.001**
Pulmonary embolism	4 (3.1)	1 (0.7)	0.21
Required oxygen support	95 (74.8)	12 (9.4)	**<0.001**
CPAP	29 (22.8)	7 (5.5)	**<0.001**
Invasive mechanical ventilation	32 (25.2)	0 (0)	**<0.001**
**Therapeutic procedures**—**no. (%)**			
HCQ	104 (81.9)	11 (8.6)	**<0.001**
HCQ + lopinavir/ritonavir	88 (69.2)	6 (4.6)	**<0.001**
HCQ + tocilizumab + lopinavir/ritonavir	31 (24.4)	3 (2.3)	**<0.001**
Glucocorticoid	48 (37.7)	13 (10.2)	**<0.001**
Antibiotic therapy	89 (70.1)	22 (17.3)	**<0.001**
**Outcome**—**no. (%)**			
Dead	32 (25.1)	0 (0)	**<0.001**

Abbreviations: CPAP, Continuous positive airway pressure; HCQ, Hydroxychloroquine; ICU, Intensive care unit.

### 3.4 Predictors of in‐hospital, all‐cause mortality in COVID‐19

We matched the thalassaemia patients with COVID‐19 patients in absence of thalassaemia to assess for predictors of in‐hospital, all‐cause mortality. In the univariate analysis, male gender, age, thalassaemia, pulmonary disease, cardiac disease, kidney disease and diabetes were all found to predict all‐cause mortality. Using a multivariable logistic regression analysis (Table 4), age (OR 1.04; 95%CI 1.04–1.04; *p* < 0.01), heart disease (OR 1.96; 95%CI 1.09–3.51; *p* = 0.02) and kidney disease (OR 4.85; 95%CI 1.02–23.0; *p* = 0.04) were revealed as independent predictors of increased in‐hospital, all‐cause mortality, whereas a history of thalassaemia (OR 0.01; 95%CI 0.008–0.021; *p* < 0.01) was revealed as an independent predictor of reduced all‐cause mortality compared to patients with no history of thalassaemia.

**TABLE 4:** Multivariable logistic regression analysis for the compliance

	Univariate analysis			Multivariable analysis		
	OR	95%CI	*p*‐value	OR	95%CI	*p*‐value
Male	0.64	0.50–0.83	**<0.01**	0.73	0.53–1.00	0.05
Age	1.05	1.04–0.1.05	**<0.01**	1.04	1.04–1.05	**<0.01**
Thalassaemia	0.01	0.007–0.015	**<0.01**	0.01	0.008–0.021	**<0.01**
Pulmonary disease	2.56	1.61–4.07	**<0.01**	1.58	0.93–2.70	0.09
Cardiac disease	3.14	1.99–4.93	**<0.01**	1.96	1.09–3.51	**0.02**
Renal insufficiency	8.40	2.08–33.93	**<0.01**	4.85	1.02–23.0	**0.04**
Hepatopathy	0.66	0.38–1.15	0.14			
Diabetes mellitus	2.51	1.58–3.98	**<0.01**	1.24	0.70–2.19	0.44

Abbreviation: OR, Odds ratio.

## 4 DISCUSSION

This present study examined patient characteristics, in‐hospital complications and outcomes of COVID‐19 patients with and without a history of thalassaemia. The main findings of our study were as follows: (1) Thalassaemia was not associated with an increased risk of mortality or need of respiratory support; (2) COVID‐19 infection in the thalassaemia cohort was generally mild and not associated with an increased risk of in‐hospital complications; (3) Thalassaemia is an independent predictor of reduced in‐hospital, all‐cause mortality compared to patients with no history of thalassaemia.

To date, various co‐factors have been identified as being associated with increased mortality in COVID‐19 patients, amongst these cancer and hemoglobinopathies.^6^ With regard to thalassaemia, the data from a small number of case series and case reports have been inconclusive. A multicentre Iranian registry investigating 23 cases of beta‐thalassaemia published a COVID‐19 mortality rate of 26%. In this Iranian cohort, the splenectomy rate was at least 80%.^11^ On the other hand, preliminary data from 11 thalassaemia patients from different Italian centres hinted at milder outcomes in COVID‐19 disease with all patients recovering completely.^13^ A different study from centres in 10 different countries summarized data from at least 10 beta‐thalassaemia patients. In this study, one patient (10%) died despite respiratory support and intensive care unit stay.^8^ Only 40% of these patients had a history of splenectomy. The mortality rate of 0.7% in our study seems to be very low in comparison, but included a far greater sample size. Approximately 40% of patients in our study had a history of splenectomy. This is comparable to the recently published study, however, with a lower mortality rate.^13^

We compared data from thalassaemia patients with COVID‐19 to a matched cohort of COVID‐19 patients with no history of thalassaemia and, seemingly paradoxically, found a significantly lower in‐hospital, all‐cause mortality rate in the thalassaemia cohort and a lower rate of in‐hospital complications. Although the rate of liver disease was higher in thalassaemia patients compared to the age‐, gender‐ and cardiovascular disease‐matched group of COVID‐19 patients, the mortality rate was much lower. Of note, recently published data described a worse prognosis in patients with liver disease, and reported a mortality rate of approximately 15%.^17^ Despite the higher prevalence of liver disease in our thalassaemia cohort compared to patients with no history of thalassaemia, the outcomes were still better in patients with thalassaemia. In a nationwide Iranian cohort, 36.4% had a heart and liver iron overload. Our present data showed an overload of iron in the heart and liver in 6.8% and 35%.

These data appear to confirm a lower risk of mortality in thalassaemia patients. In our present study cohort, 99% of patients recovered from COVID‐19.

Whereas the need for intubation was documented in only one patient, non‐invasive respiratory support was required in seven patients in our study. Compared to the matched cohort, however, these rates were still significantly lower than in the non‐thalassaemia group. The mild courses of COVID‐19 in the thalassaemia cohort may be related to different factors. Recently published data showed that ORF8 and surface glycoproteins of COVID‐19 could form a complex together with porphyrin. In addition, orflab, ORF10 and ORF3a proteins could attack the porphyrin, which is formed in the mitochondria on the 1‐beta chain of haemoglobin.^18^, ^19^ This may lead to dissociation of iron from porphyrin. Genetic mutations may result in the absence of beta chain synthesis while retaining normal alpha chain synthesis. This could overall lead to less haemoglobin A being produced. Since the beta chain s a potential target for COVID‐19 infection is either absent or expressed at a much lower rate in the blood of thalassaemia patients, this may lead to decreased susceptibility to COVID‐19 infection in thalassaemia patients.^17^ Of note, a history of regular blood transfusions was documented more than 80% of our thalassaemia patients. But nevertheless, in vitro and animal studies are required to confirm this hypothesis. It might be speculated that in transfusion‐dependent patients when pretransfusion haemoglobin goal is 9–10 g/dl, their bone marrow is supposed to be suppressed by transfusions therefore the majority of haemoglobin A is from normal transfused red cells and less produced by patients own abnormal red blood cells. It could be that protecting red cells from COVID‐19 will be irrelevant in TDT patients but could apply to TIT patients.

The majority of patients in our cohort came from European countries and as a result, ethnic differences regarding their outcomes cannot be excluded. In the present study, it seems that transfusion independent thalassaemia patients are suffering more significantly from acute kidney injury and they need more oxygen support. Although invasive and non‐invasive respiratory support and mortality are numerically higher, not a significant difference was documented.

A recent observational study regarding the relevance of patients’ blood groups and the risk of COVID‐19 infection reported that patients with blood group A are at a higher risk to develop COVID‐19 and patients with blood group O are at a lower risk for developing COVID‐19.^20^ The Iranian study on thalassaemia reported a low frequency (13%) of blood group O.^11^ In the 10 cases published by Sanctis et al., no patients had blood group O. Our data however showed a high frequency of blood group O. This may confirm the multifactorial role in the outcome of COVID‐19 patients.

Differences with regard to the treatments used were also observed between groups. Drugs and drug combinations such as hydroxychloroquine, hydroxychloroquine + lopinavir + ritonavir or hydroxychloroquine + tocilizumab + lopinavir + ritonavir were more likely to have been used in the COVID‐19 with no history of thalassaemia, which might simply be reflecting the milder courses of COVID‐19 infection observed in thalassaemia patients.

Compared to the matched cohort, COVID‐19 symptoms were significantly lower amongst thalassaemia patients.

In the Iranian thalassaemia registry, the prevalence of COVID‐19 was 0.14% in the thalassaemia patients. On this point, we cannot provide any data regarding the prevalence of COVID‐19 in thalassaemia patients since we included all patients with COVID‐19 and did not analyse the number of thalassaemia patients in each and every centre.

## 5 CONCLUSIONS

Analysis of the data of this large international cohort revealed a lower all‐cause mortality rate in thalassaemia patients compared to a matched cohort with no history of thalassaemia. In‐hospital complications relating to COVID‐19 infection were also significantly lower in thalassaemia patients, although the hospitalization rate was 27.7%, which was significantly lower than in the matched cohort.

### 5.1 Limitations

Data for this study were collected from several centres and bias regarding the heterogeneous diagnostic and treatment strategies between them cannot be excluded. In addition, there are differences in the capacity and other medical resources available in these centres. Although the data summarized in this study came from an international registry, the majority of patients included were from European countries (Italy and Spain) and outcomes may differ due to ethnic factors and standard of care in other parts of the world.

## CONFLICT OF INTEREST

The authors declare no conflicts of interest.

## AUTHOR CONTRIBUTION

**Ibrahim El‐Battrawy:** Conceptualization (equal); Data curation (equal); Formal analysis (equal); Supervision (equal); Validation (equal); Visualization (equal). **Filomena Longo:** Data curation (equal); Formal analysis (equal). **Iván J. Núñez‐Gil:** Conceptualization (equal); Data curation (equal); Formal analysis (equal); Supervision (equal). **Mohammad Abumayyaleh:** Data curation (equal); Formal analysis (equal). **Barbara Gianesin:** Data curation (equal); Formal analysis (equal). **Vicente Estrada:** Data curation (equal). **Alvaro aparisi:** Data curation (equal). **Ramón Arroyo‐Espliguero:** Data curation (equal). **Manuela Balocco:** Data curation (equal); Formal analysis (equal). **Susanna Barella:** Data curation (equal). **Andrea Beccaria:** Data curation (equal). **Federico Bonetti:** Conceptualization (equal); Data curation (equal). **Maddalena Casale:** Data curation (equal). **Elisa De Michele:** Investigation (equal). **AnnaRita Denotti:** Data curation (equal); Software (equal). **Carmelo Fidone:** Data curation (equal). **Monica Fortini** **:** Formal analysis (equal); Investigation (equal). **Maria Rita Gamberini:** Data curation (equal). **Giovanna Graziadei:** Formal analysis (equal). **Roberto Lisi:** Data curation (equal). **Antonella Massa:** Data curation (equal). **Alessia Marcon:** Data curation (equal). **Bryan Rubinski:** Investigation (equal); Writing‐review & editing (equal). **Maurizio Miano:** Formal analysis (equal); Methodology (equal). **Irene Motta:** Software (equal); Supervision (equal). **Valeria Pinto:** Validation (equal); Visualization (equal). **Alberto Piperno:** Supervision (equal); Visualization (equal). **Raffella Mariani:** Methodology (equal); Visualization (equal). **Maria Caterina Putti:** Visualization (equal). **Alessandra Quota:** Software (equal); Supervision (equal). **Michela Ribersani:** Data curation (equal). **Marco Marziali:** Data curation (equal). **Domenico Roberti:** Formal analysis (equal). **Rosamaria Rosso:** Software (equal). **Immacolata Tartaglione:** Data curation (equal). **Angelantonio Vitucci:** Data curation (equal). **Vincenzo Voi:** Data curation (equal). **Marco Zecca:** Data curation (equal); Formal analysis (equal). **Rodolfo Romero:** Data curation (equal); Formal analysis (equal). **Charbel Marouneld:** Methodology (equal). **Inmaculada Fernández‐Rozas:** Data curation (equal). **Carolina Espejo:** Investigation (equal). **Wulandewi Marhaeni:** Investigation (equal). **Marcos Garcia Aguado:** Data curation (equal). **Maria Domenica Cappelini:** Software (equal). **Silverio Perrotta:** Data curation (equal). **Lucia De Franceschi:** Data curation (equal). **Antonio Piga:** Formal analysis (equal). **Gian Luca Forni:** Supervision (equal). **Ibrahim Akin:** Validation (equal); Visualization (equal); Writing‐original draft (equal); Writing‐review & editing (equal).

## Supporting information

Table S1

## Data Availability

Raw data are available on request by the correspoinding author
