# Supplementary material for: Thalassaemia is paradoxically associated with a reduced risk of in‐hospital complications and mortality in COVID‐19: Data from an international registry
Source: J Cell Mol Med. 2022 Mar 30;26(9):2520–8. doi: 10.1111/jcmm.17026 (PMC9077285; doi:10.1111/jcmm.17026)
Supplement: Supplementary file 1 — Table S1 [file JCMM-26-2520-s001.docx]

| Table S1. Characteristics of TIT as compared to TDT patients at Baseline, complication, therapeutic procedures, and outcome. | | | |
| --- | --- | --- | --- |
| Characteristic | TDT  n=112 | TIT  n=22 | P value* |
| Age ––yr mean ±SD ç | 38±14 | 54.7±18.6 | **<0.001** |
| Male sex ––no. (%) | 52/112 (46.4) | 13/22 (59.1) | 0.28 |
| **Blood Group ––no. (%)** |  |  |  |
| A Rh- | 2/100 (2) | 0/9 (0) | 1.00 |
| A Rh+ | 34/100 (34.4) | 3/9 (33.3) | **<0.001** |
| AB Rh+ | 3/100 (3) | 0/9 (0) | - |
| B Rh+ | 13/100 (13) | 1/9 (11.1) | **<0.001** |
| 0 Rh- | 4/100 (4) | 0/9 (0) | 1.00 |
| 0 Rh+ | 44/100 (44) | 5/9 (55.6) | **<0.001** |
| **Iron load** |  |  |  |
| Serum Ferritin mg/l (median(min-max)) | 719 (27-8140) | 567 (42-4057) | 0.34 |
| Liver MRI T2 ms (mean±SD) | 11.4±7.5 | 6.2±2.5 | **0.001** |
| Heart MRI T2 ms (mean±SD) | 33.4±10 | 44.3±6.6 | **0.04** |
| LIC (mg Fe/g d.w.) (median(min-max)) | 2.6 (1.1-127.2) | 4.8 (3.6-7.8) | 0.83 |
| Hepatic iron overload ––no. (%) | 24/73 (32.9) | 4/7 (57.1) | 0.23 |
| Cardiac iron overload ––no. (%) | 5/70 (7.1) | 0/4 (0) | 1.00 |
| **Therapy ––no. (%)** |  |  |  |
| **Chelation therapy** |  |  |  |
| DFO | 14/105 (13.3) | 2/12 (16.7) | **<0.001** |
| DFP | 19/105 (18.1) | 1/12 (8.3) | **<0.001** |
| DFX | 57/105 (54.3) | 2/12 (16.7) | **<0.001** |
| DFO+DFX | 2/105 (1.9) | 0/12 (0) | 1.00 |
| DFP+DFO | 7/105 (6.7) | 0/12 (0) | 1.00 |
| DFP+DFX | 3/105 (2.9) | 0/12 (0) | 1.00 |
| no | 3/105 (2.9) | 7/12 (58.3) | **<0.001** |
| **HU therapy** | 2/88 (2.3) | 4/13 (30.8) | **0.002** |
| Hospitalized | 23/112 (20.5) | 12/22 (54.5) | **0.001** |
| ICU Admission ––no. (%) | 20/112 (17.9) | 5/22 (22.7) | 0.59 |
| **Chronic conditions –– no. (%)** |  |  |  |
| Diabetes Mellitus | 13/112 (11.6) | 1/22 (4.5) | 0.47 |
| Obesity | 6/112 (5.4) | 5/22 (22.7) | **0.007** |
| Renal insufficiency | 5/112 (4.5) | 2/22 (9.1) | 0.32 |
| Lung disease | 9/112 (8) | 2/22 (9.1) | 1.00 |
| Cardiac disease | 22/122 (19.6) | 4/22 (18.2) | 1.00 |
| Pulmonary hypertension | 3/108 (2.8) | 2/15 (13.3) | 0.11 |
| Liver disease | 16/112 (14.3) | 3/22 (13.6) | 1.00 |
| Previous HCV hepatitis | 3/108 (2.8) | 0/15 (0) | 1.00 |
| Hypothyroidism | 17/112 (15.2) | 2/22 (9.1) | 0.74 |
| Hypogonadism | 17/108 (15.7) | 0/15 (0) | 0.13 |
| Osteoporosis | 30/108 (27.8) | 1/15 (6.7) | 0.11 |
| G6PD deficiency | 4/108 (3.7) | 0/14 (0) | 1.00 |
| Splenectomy | 46/112 (41.1) | 4/15 (26.7) | 0.40 |
| **Symptomatic –– no. (%)** |  |  |  |
| Dyspnea | 16/112 (14.3) | 9/22 (40.9) | **0.003** |
| Rhinorrhea | 26/112 (23.2) | 3/22 (13.6) | 0.41 |
| Fatigue | 14/112 (12.5) | 10/22 (45.5) | **<0.001** |
| Anosmia / Dysgeusia | 32/112 (28.6) | 3/22 (13.6) | 0.19 |
| Pain | 41/112 (36.6) | 7/22 (31.8) | 0.67 |
| Headache | 29/112 (25.9) | 4/22 (18.2) | 0.59 |
| Sorethroat | 32/112 (28.6) | 3/22 (13.6) | 0.19 |
| Fever | 50/112 (44.6) | 17/22 (77.3) | **0.005** |
| Cough | 44/112 (39.3) | 16/22 (72.7) | **0.004** |
| Diarrhea | 10/112 (8.9) | 3/22 (13.6) | 0.45 |
| X-Ray abnormality –– no. (%) | 13/25 (52) | 9/12 (75) | 0.29 |
| CT abnormality –– no. (%) | 11/19 (57.9) | 4/4 (100) | 0.26 |
| **Complications and procedures –– no. (%)** |  |  |  |
| Anemia | 5/5 (100) | 4/7 (57.1) | 0.21 |
| Acute kidney injury | 0/112 (0) | 5/22 (22.7) | **<0.001** |
| Pulmonary embolism | 1/122 (0.9) | 0/22 (0) | 1.00 |
| Required oxygen support | 6/112 (5.4) | 7/22 (31.8) | **<0.001** |
| CPAP | 5/112 (4.5) | 2/20 (10) | 0.29 |
| Invasive mechanical ventilation | 0/112 (0) | 1/21 (4.8) | 0.16 |
| **Therapeutic procedures –– no. (%)** |  |  |  |
| HCQ | 0/112 (0) | 8/21 (38.1) | **<0.001** |
| Interleukin-1 receptor antagonist | 1/112 (0.9) | 0/15 (0) | 1.00 |
| Lopinavir/ritonavir | 5/112 (4.5) | 2/20 (10) | 0.29 |
| HCQ, tocilizumab and lopinavir/ritonavir | 1/112 (0.9) | 1/20 (5) | 0.28 |
| Glucocorticoid | 7/112 (6.3) | 6/20 (30) | **0.001** |
| Antibiotic therapy | 12/112 (10.7) | 10/21 (47.6) | **<0.001** |
| LMWH | 5/112 (4.5) | 8/21 (38.1) | **<0.001** |
| Remdesivir | 2/112 (1.8) | 1/15 (6.7) | 0.32 |
| Blood transfusion | 5/112 (4.5) | 2/15 (13.3) | 0.19 |
| MEEX | 1/108 (0.9) | 0/14 (0) | 1.00 |
| **Outcome –– no. (%)** |  |  |  |
| Dead | 0/85 (0) | 1/22 (4.5) | 0.19 |

Abbreviations: TIT, Transfusion independent thalassemia; TDT, transfusion dependent thalassemia; MRI, magnet resonance imaging; LIC, liver iron concentrations; DFO, deferoxamine; DFP, deferipone; DFX; deferasirox; HU, hydroxyurea; IgG, immunoglobulin g; ICU, intensive care unit; HCV, hepatitis virus C; G6PD, glucose-6-phosphate dehydrogenase; CT, computer tomography; CPAP, continuous positive airway pressure; HCQ, hydroxychloroquine; LMWH, low-molecular-weight heparin; MEEX, manual erythron-exchange.
